# EXD2 - a new player joins the DSB resection team

**DOI:** 10.1080/15384101.2016.1161997

**Published:** 2016-05-31

**Authors:** Jadwiga Nieminuszczy, Ronan Broderick, Wojciech Niedzwiedz

**Affiliations:** Department of Oncology, Weatherall Institute of Molecular Medicine, University of Oxford, Oxford, OX3 9DS, UK

**Keywords:** DNA double strand break, DNA-end resection, EXD2, homologous recombination, MRE11

DNA double-strand breaks (DSBs), i.e. where both strands of the DNA double helix are broken, are among the most toxic type of damage that cells can suffer. They can arise during normal cellular processes or are induced by commonly used anti-cancer modalities, such as ionising radiation. Unrepaired DSBs can result in cell death, and their miss-repair drives genome rearrangements and the loss of genetic information at the break site. Therefore their error-free repair is essential not only for cell survival, but also for organismal development, as mutations in genes involved in this process underline various inherited human syndromes characterized by predisposition to cancer, immunodeficiency and premature aging.^1^ However, despite their importance to genomic stability and their role in anti-cancer therapy, the mechanisms behind DSB repair are not fully understood.

The two major pathways involved in the repair of DSBs in eukaryotic cells are the error prone non–homologous end-joining (NHEJ), that involves the ligation of broken DNA ends (which often results in the loss of genetic information), and an error free process called homologous recombination (HR) that utilises the intact DNA template of the undamaged sister chromatid. HR is particularly important for repairing DSBs arising in S-phase due to replication fork collapse, during which NHEJ can be highly dangerous as it generates oncogenic genome rearrangements.^2^

A key initial step in HR is resection of the DNA ends on either side of the DSB, which until now has been thought to be carried out by the MRE11-RAD50-NBS1 complex (MRN) and CtIP, resulting in generation of short stretches of single stranded DNA (ssDNA). Subsequently, the EXO1 or DNA2 nucleases, in conjunction with the Bloom's syndrome helicase (BLM) extend these to generate longer 3′ ssDNA tails that are bound by RPA. Replacement of RPA by RAD51, in a BRCA2-dependent manner, leads to the formation of ssDNA-RAD51 nucleoprotein filaments essential for strand exchange and homology directed repair. Interestingly, inhibition of MRE11 endonuclease activity confers a stronger resection defect than inhibition of its exonuclease activity, suggesting perhaps that other nucleases might be involved in the initial break processing.^3^ In line with this, recent work from our laboratory identified EXD2 as a novel 3′-5′ exonuclease and cofactor of the MRN complex, which is required for efficient DNA-end resection.^4^

So what is the relative contribution of EXD2 to the process of DNA-end resection? To address this we used the intensity of RPA foci at different time points (ref^4^ and Figure 6a within) to estimate the kinetics of resection in WT and EXD2 depleted cells exposed to ionising radiation. We assumed that RPA loading on ssDNA correlates with the speed of resection. Thus, the slope of the line of best fit could be used as an indicator of “relative resection rate.” This analysis shows that in the absence of EXD2 DNA-end resection is reduced to about 30% of the rate observed in WT cells (slope 0.56 for WT and 0.18 for EXD2-depleted cells). This is interesting from a mechanistic point of view, as together with data presented in ref.^4^ it suggests that in vertebrates EXD2 could be the main 3′-5′ exonuclease required for initial DNA end-processing.

This begs the question: what would be the benefits of accelerated resection during DSB processing? One possibility is that the kinetics of resection influences DSB repair pathway choice. For example, slower initial kinetics of resection could favor error-prone repair through single strand annealing (SSA) pathway and/or NHEJ/A-NHEJ, which ultimately may result in genome rearrangements. Accordingly, short homologous segments favor error-prone SSA in yeast.^6^ Moreover, Drosophila melanogaster EXD2-mutants and EXD2-deficient U2OS cells display spontaneous genome instability.^4,5^ Another possibility, not mutually exclusive, is that EXD2 degrades damaged (modified) DNA templates, which otherwise would be inhibitory to MRE11-dependent resection. EXD2 alone or in collaboration with the MRN complex could also participate in the removal of protein bound to DNA-ends (Model Fig. 1).

**Figure 1.**
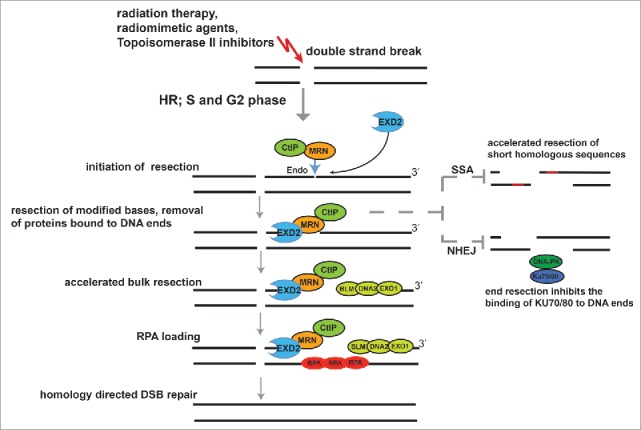
A model for EXD2′s role in suppressing genome instability. EXD2 accelerates DNA-end resection initiated by the MRN/CtIP complex. Subsequently, EXO1 or DNA2, in conjunction with BLM generate longer 3′ ssDNA tails. RPA loaded on ssDNA is then exchanged for RAD51 to promote strand invasion and HR. Processed DSB-ends are no longer suitable substrates for SSA or NHEJ.

Recently, homologous recombination has emerged as an important target in cancer treatment because cancer cells rely heavily on HR for repair of damaged DNA. Thus, inhibition of HR may enhance the toxicity of many commonly used anti-cancer drugs or it may also be useful as a monotherapy to selectively kill cancer cells defective in redundant repair pathways. In this regard, disruption of EXD2′s exonuclease activity induces chemo- and radiosensitization ^4^ (and data not shown). Therefore, it is conceivable that development of small molecule inhibitors targeting EXD2′s exonuclease activity may allow for synergistic treatments with existing chemo- or radiotherapeutic approaches, improving patient survival and increasing the therapeutic window. Likewise, expression of MRE11 was recently shown to be a predictive biomarker of cause-specific survival following radical radiotherapy for muscle-invasive bladder cancer.^7^ This follows that EXD2 expression level could offer similar benefits as a biomarker in guiding patients' stratification for the most effective therapy.

In summary, a complete understanding of the mechanism of HR is of great importance in clinical oncology, where there are major efforts to increase the efficacy of DNA-damaging agents. In light of this, EXD2 with its exonuclease activity constitutes an exciting, hitherto largely unexploited target for developing novel therapeutic strategies to treat cancer.

## References

[1] Jackson SP, et al. Nature 2009; 461:1071-8; PMID:19847258; http://dx.doi.org/10.1038/nature08467 19847258 PMC2906700

[2] Jasin M, et al. Cold Spring Harbor Perspectives Biol 2013; 5:a012740; http://dx.doi.org/10.1101/cshperspect.a012740 PMC3809576 24097900

[3] Shibata A, et al. Mol Cell 2014; 53:7-18; PMID:24316220; http://dx.doi.org/10.1016/j.molcel.2013.11.003 24316220 PMC3909494

[4] Broderick R, et al. Nat Cell Biol 2016; 18(3):271-80.; PMID:26807646 26807646 10.1038/ncb3303 PMC4829102

[5] Saunders RD, et al. Aging Cell 2008; 7:418-25; PMID:18346216; http://dx.doi.org/10.1111/j.1474-9726.2008.00388.x 18346216 PMC2408639

[6] Sugawara N, et al. Mol Cell Biol 2000; 20:5300-9; PMID:10866686; http://dx.doi.org/10.1128/MCB.20.14.5300-5309.2000 10866686 PMC85979

[7] Choudhury A, et al. Cancer Res 2010; 70:7017-26; PMID:20843819; http://dx.doi.org/10.1158/0008-5472.CAN-10-1202 20843819 PMC2941719

